# Mandarin Stroke Social Network Scale and Item Response Theory

**DOI:** 10.3389/fstro.2022.903289

**Published:** 2022-08-11

**Authors:** Chun Feng, Qing-Ling Lai, Amanda Ferland, Feng Lin

**Affiliations:** ^1^The Center of Rehabilitation Therapy, The First Rehabilitation Hospital of Shanghai, Rehabilitation Hospital Affiliated to Tongji University, Shanghai, China; ^2^Department of Rehabilitation Medicine, The First Hospital of Quanzhou Affiliated to Fujian Medical University, Quanzhou, China; ^3^Center of Rehabilitation Medicine, The First Affiliated Hospital of Nanjing Medical University, Nanjing, China

**Keywords:** Stroke Social Network Scale, questionnaire design, reliability and validity, perceived social support, Item Response Theory

## Abstract

**Background:**

Stroke survivors may have difficulty in social integration due to physical or mental disorders across the entire continuum of disease. Higher perceived social support can promote social participation for persons following a stroke. However, no scale is available to investigate the perceived social support among the Chinese post-stroke population.

**Objectives:**

The current study addresses this gap by developing the Mandarin version of the Stroke Social Network Scale (M-SSNS) and examining the reliability and validity of this scale. This study also utilizes the Item Response Theory (IRT) model as a bridge between social ability and functional status.

**Methods:**

The convenience sampling method was followed to recruit 71 inpatient post-stroke individuals. All participants were evaluated by the modified Barthel Index (MBI), M-SSNS, and the Extended *International Classification of Functioning, Disability, and Health* (ICF) Core Set for Stroke. The reliability of M-SSNS was explored based on the IRT model. The validity of the scale was further confirmed by assessing the correlation between estimated personal social competence and the final score of the 19-item M-SSNS, and the correlation between estimated personal social competence and functional status based on the ICF.

**Results:**

A total of 71 patients with stroke (53 males and 18 females) were included in this research. Fourteen items of M-SSNS were selected to represent personal social ability according to the unidimensional 3-parameter logistic (3PL) IRT model. The Cronbach's α of the 14-item scale was 0.7192, with the Guttman's λ_2_ = 0.7567, Molenaar Sijtsma ρ = 0.7491, and latent class reliability coefficient = 0.8657, indicating that the M-SSNS had great internal consistency. The estimated individual social competence by the 14-item 3PL model was highly related to the final score of the 19-item M-SSNA (*p* < 0.001, *r* = 0.79). The correlation between the personal functional status and social ability was 0.23 (*p* = 0.049, *r* = 0.23).

**Conclusion:**

The 14-item M-SSNS manifests great reliability and acceptable validity. Based on the IRT, the 14-item M-SSNS is also a promising tool to assess the social structure and provide customized relationship consulting, education, and advice among the Chinese stroke population.

## Introduction

Individuals after stroke appear to suffer from social isolation due to physical and/or mental deficits (Sun et al., 2014; Virani et al., 2020). According to survivors' self-report, 72% of individuals after stroke report deterioration of their social participation (Mayo et al., 2002), and their social networks can be susceptible to shrinking. Although participation might have diverse meanings to different people, Eriksson et al. (2013) suggest that the ability to participate in previous activities, home and community engagement, and good perception of stroke recovery are the three main factors for successful participation in this population.

Although China has the world's largest population of people who have had a stroke (Wang et al., 2017), there is no available scale related to social participation and social network assessment for people who have had a stroke in China. The Chinese version of Stroke and Aphasia Quality of Life Scale-39 (Qiu et al., 2019) evaluates the quality of life for the post-stroke population with aphasia in China, but may not directly reflect their social reintegration. The Medical Outcomes Studies Social Support Survey investigates limitations in social participation but does not consider the overall social structure (Thompson et al., 2014). Additionally, it has not been examined for its reliability and validity for the stroke population on the Chinese mainland (Yu et al., 2004, 2015). The Chinese version of the Language Screening Test focuses on language function, rather than how language ability affects social interaction (Yang et al., 2018). As one of the few stroke social structure assessments, the Stroke Social Network Scale (SSNS), first developed by Northcott and Hilari, aims to differentiate between those with high vs. low perceived social support (Northcott and Hilari, 2013). It has been proved to provide a standardized test for stroke patients with great heterogeneity of functional status. Moreover, the SSNS is a reliable and valid scale based on self-report, even for patients with speech difficulties.

The next few questions were also considered for our study. How do we measure the social ability deterioration that the stroke population is experiencing? Which social roles are the most difficult to participate in, and/or which relationship is most affected for certain stroke individuals?

Scholars have introduced the Item Response Theory (IRT) to measure personal ability and differentiate item difficulties (Hays et al., 2000). Moreover, Schalet et al. (2021) suggest that the IRT model can facilitate understanding of the association between the patient-report measures and functional assessment scales, and provide evidence for clinical decision-making. In recent years, non-parametric IRT models based on the Mokken scale analysis (MSA) have begun to attract attention in the medical areas (Stochl et al., 2012; Thompson et al., 2014; Vaughan and Grace, 2018; Zhang and Li, 2020). Rather than following strict rules for the parametric IRT, MSA can offer preparation processes of data shaping and hypothesis testing before parametric IRT modeling (Lee et al., 2016; Feng et al., 2022).

The purpose of this study was to investigate the psychometric properties of the Mandarin version of SSNS (M-SSNS) among the Chinese stroke population. Considering the context of the culture and value systems, the perception of social support and stroke outcomes might also differ (Guo et al., 2016; Lawal et al., 2020). It is necessary to adjust the SSNS to form a Mandarin version based on Chinese culture and socioeconomic background. We also attempted to exploit personal social competence and the difficulty level of maintaining diverse social relationships based on the IRT model. We assumed that the M-SSNS was an effective scale for assessing social networks for post-stroke Chinese persons. The participation limitation could be targeted to intervene based on the IRT model in future practice.

## Methods

A cross-sectional design employed in this study was approved by the Ethical Committee of Shanghai local rehabilitation hospital. Given the maximum variation sampling strategy, this study recruited 71 patients hospitalized in the rehabilitation hospital setting, involving acute, subacute, or chronic phases of recovery from stroke by convenience sampling from January 2021 to January 2022.

### The features of the original SSNS

The Stroke Social Network Scale (SSNS) has 19 items and emphasizes the relationship with children, friends, relatives, neighbors, and the community. The scale also concerns the size of the network, the composition of the network, frequency of contact, proximity, and satisfaction with the network.

The six domains can also be combinedly or separately analyzed including four items for children (C), three items for relatives (R), four items for friends (F), two items for the community (WN), five items for satisfaction (S), and one item for loneliness (L). The children's domain covers four similar questions to the friend's domain, involving the number of child/friends, frequency of seeing child/friend in person, the frequency of contacting child/friend with telecommunications, and distance away from child/friend. The satisfaction domain includes patient-report satisfaction with the child, relatives, close friends, neighbors, and overall social network. The score of each item is from 0 to 100. For example, item C2 “*In the past month, how often did you see your children?”* contains response option as “not at all = 0; about once a month = 20; 2 or 3 times a month = 40; at least once a week = 60; 2 or 3 times a week = 80; every day = 100.” The final score of the 19-item SSNS is the mean score of all items. The final scores range from 0 to 100. Patients who score lower in the SSNS indicate few social ties and less social support.

### Translation and cross-cultural adaptation of M-SSNS

Two native Mandarin translators independently translated the English version to Chinese after permission from Northcott and Hilari (2013). Any ambiguities and discrepancies were addressed by consulting the original authors of the SSNS.

Due to the differences in culture and communication tools between China and the Western world, communication habits, especially recently, have diverged. For example, the “WeChat” APP is used extensively and almost exclusively in China, and it has substantially impacted interpersonal communication behavior (Montag et al., 2018). However, the C3 “*In the past month, how often were you in contact with your children by telephone, letter or email?”* in the original scale could not be indicative of common communication tools for most Chinese people; therefore, it was adjusted to “*In the past month, how often were you in contact with your children by telephone, WeChat (Wēixìn), Email or other ways?”* The same adjustment was made to R3 and F3. The final version of the M-SSNS was then constructed after these revisions. The final Mandarin version of SSNS can be retrieved from Supplementary 1.

### Participants

The eligibility criteria included (Northcott and Hilari, 2013): (1) Diagnosis of stroke by Computed tomography or Magnetic Resonance Imaging; (2) Age≥18 years; (3) Able to provide informed consent. The exclusion criteria included: (1) Unhealed trauma or surgical incision; (2) Patients with critical illness, such as severe cardiopulmonary failure; (3) Other diseases affecting data collection, such as a history of mental illness or severe dementia.

### Procedures

After providing written informed consent, participants were asked to complete a 1-h interview and clinical examination in a private room. The demographic information collected included gender, age, and concomitant disorders, as well as education levels. The stroke type, involved side, and the course of the stroke were reviewed through patient medical charts or face-to-face interviews.

One trained evaluator evaluated 19-item M-SSNS as social network analysis. Functional ability was also assessed using the MBI and Extended *International Classification of Functioning, Disability, and Health (ICF)* core set for stroke (Starrost et al., 2008). The collection process was supervised by another examiner to control the inter-rater reliability.

The ICF is designed to describe human experience regarding three main health outcomes, Impairment (I), Activity Limitation (A), and Participation Restriction (P) (World Health Organization, 2013). The ICF qualifier scale is a 5-point Likert scale with numeric rating ranks “no impairment” = 0; “mild impairment” = 1, “moderate impairment” = 2, “severe impairment” = 3, “complete impairment” = 4 (Geyh et al., 2004). The qualifiers of environmental factors were marked as “-1,−2,−3,−4” to indicate their hindering impact, and “+1, +2, +3, +4” to indicate their facilitating impact.

Extended ICF Core Set for Stroke consists of 166 items (Starrost et al., 2008), including 59 items related to body function (b), 59 items related to activity and participation (d), 37 items related to environmental and individual factors (e), and 11 items related to body structure (s). For studying the construct validity of the SSNS, we sorted out the b and d categories and excluded the e and s categories. The main reason for excluding e categories in the inpatient setting is due to the fact that this information includes the individual attitudes of personal care providers, health professionals, and other professionals. It would bias this aspect of the scoring because of admission to the hospital. In addition, the qualifier of e categories is scored differently than b, d, and s. The s category with non-interventional items, such as the structure of the brain, were also excluded. The b and d categories were chosen to provide stroke-related activity and participation items.

### Data processing

The software R version 4.1.0 was applied for all the computations. Descriptive statistics of mean and standard deviation were used to describe the demographical data. The education time, stroke types, involved side, age, the course of the stroke, concomitant disorders, and scores of MBI were tested across gender with χ^2^ test for categorical data and *t*-test for continuous data.

To explore the reliability of the scale, the R-package “mokken” and “mirt,” as well as the guideline of Sijtsma and van der Ark (Sijtsma and van der Ark, 2017), were used for MSA and Rasch model. The Pearson correlation coefficient was calculated between estimated personal social competence and final score of the scale, and the ICF-based activity and participation items for investigating the validity of the model.

### Data shaping

**Figure 1:**
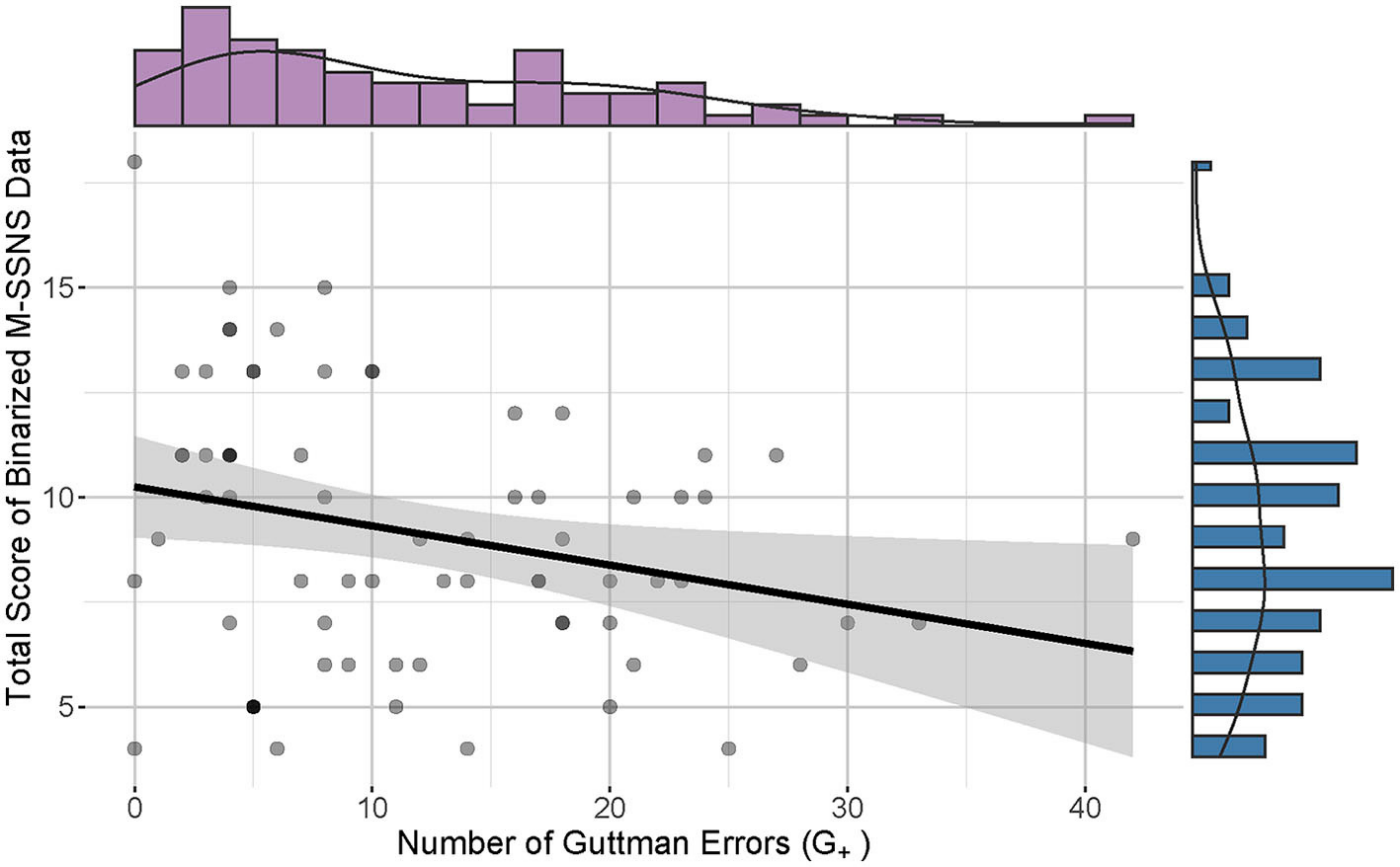
Correlation between total score M-SSNS and number of Guttman Errors (G+). The scatterplot and line graph show a slightly negative relationship between the total score of M-SSNS and the number of G+. The purple histogram displays the G+ distribution on the top and the blue bar graph reveals the distribution of the total score on the right.

The imputed data were then binarized using the following criteria: the score of single items ≥50 was counted as 1 (Satisfactory) and those with a score <50 were counted as 0 (Dissatisfactory). After dichotomizing the score, the number of Gutmann errors was calculated to further analyze the relationship between the total score of the binarized data in each subject and his/her number of Gutmann errors (G+). A G+ adjusted boxplot was displayed in Figure 1. The subjects would be deleted if the personal G+ exceeded the upper limit of G+.

### Scale formation based on the MSA and Rasch model

First, the global scalability coefficient (denoted as H) of the items was calculated to provide the discrimination power of the items (Sengul Avsar and Tavsancil, 2017). Second, the Automatic Item Selection Procedure (AISP) was utilized according to the genetic algorithms (Straat et al., 2013). The scalability coefficient boundary value was set from 0.3 to 0.54 (step length = 0.03). **Table 2** listed the distributions of scalable and unscalable items under different lower bound values. According to Straat et al. (2014), the sample size may be qualified as adequate (*N* = 50) to good (*N* = 250). To meet the minimum sample size requirements for MSA, unscalable items would be removed according to the threshold value of lower bound = 0.42. The uni- and two-dimensional IRT models with one to four parameters were considered as candidate models. These models were named “dimension_PL.” For example, the “dim1_Rasch” is the unidimensional one parameter logistic model, the “dim1_3PL” is the unidimensional three parameters logistic model, and the “dim2_3PL” is the two-dimensional three parameters logistic model. Each model was tested by using the M2 model fit statistic embedded in the “mirt” package. The models with *p* > 0.05 were of good fitness. The good fitted models were compared by the Akaike information criterion (AIC) and likelihood ratio test (χ^2^).

The local independence of pairwise items i and j were checked through Pearson χ^2^ test of residuals. The *p*-value and Cramer's V were visualized by plotting a heatmap. The pair with *p* < 0.05 and the absolute value of Cramer's V > 0.15 (Akoglu, 2018) were considered a significantly strong correlation and should be categorized as a local dependency. After screening the local independence, the items were tested for goodness of fit by root-mean-squared error analysis (RMSEA) and the S-X^2^ test. The *p-*values were adjusted by Benjamini-Hochberg multiple testing correction. The adjusted *p* > 0.05 were marked as good fitness.

### Reliability and validity of the SSNS

The reliability of the scale was then explored for internal consistency. Four reliability statistics were calculated: Cronbach's α, Guttman's λ_2_, Molenaar Sijtsma ρ, and the latent class reliability coefficient (LCRC) (Sengul Avsar and Tavsancil, 2017).

The correlation was assessed between the final score of the original M-SSNS and the personal ability level, that is, the social network competence, estimated by the IRT model of M-SSNS. The construct validity was examined by the Pearson correlation between the M-SSNS-IRT-based social ability and functional abilities of b and d components was examined according to our ICF-IRT model. In our previous work, we developed the ICF-IRT model with great reliability and validity among 130 stroke survivors, which can manifest personal functional competence and ICF item difficulty. We also revealed the process to build the ICF-IRT model in detail (Feng et al., 2022).

## Results

### Demographical information

A total of 71 patients with stroke (53 males and 18 females) were recruited for this research (Table 1). As described in the mean ± standard deviation, the age was 62.96 ± 12.62 years and disease course was 233.90 ± 541.72 days. Stoke types included 25.35% of hemorrhage, 69.01% of infraction, and 5.63% of the mixed category. The paralyzed part involved 43.66% of right plegia, 53.52% of left plegia, and 2.82% of others. MBI score was 57.62 ± 24.07. About 25.35% of participants had speech problems, 8.45% of which included swallowing problems, and 12.68% of which included speech plus swallowing problems. Considering education levels, four patients graduated from primary school (5.63%), 23 from middle school (32.39%), 15 from high school (21.13%), 3 from secondary school (4.23%), 4 from diploma school (5.63%), and 16 from college (22.54%); 5 patients held master's degrees (7.04%) and 1 patient held a PhD degree (1.41%).

**Table 1:** Demographical information.

**M(SD)/count (percent)**	**Overall**	**Male**	**Female**	**Method**	***p***	**Statistics**
	***N*** ** = 71**	***N*** ** = 53**	***N*** ** = 18**	**Male vs. female**		***t_(*df*)_ or $\chi_{\left(df\right)}^{2}$***
Age (year)	62.96 (12.62)	62.00 (12.38)	65.78 (13.27)	*t*-test	0.2978	1.06_(27.75)_
Disease Course (day)	233.90 (541.72)	253.08 (609.29)	177.44 (261.33)	*t*-test	0.4693	−0.73_(65.13)_
Modified Barthel Index (score)	57.62 (24.07)	57.58 (25.29)	57.72 (20.70)	*t*-test	0.9818	0.02_(35.62)_
**Stroke type**				Fisher test	0.6515	-
Hemorrhage	18 (25.35%)	12 (22.64%)	6 (33.33%)			
Infarction	49 (69.01%)	38 (71.70%)	11 (61.11%)			
Mixed	4 (5.63%)	3 (5.66%)	1 (5.56%)			
**Paralyzed part**				Fisher test	0.3757	-
R	31 (43.66%)	22 (41.51%)	9 (50.00%)			
L	38 (53.52%)	30 (56.60%)	8 (44.44%)			
Other	2 (2.82%)	1 (1.89%)	1 (5.56%)			
**Concomitant disorder**				Fisher test	0.1007	-
Speech	18 (25.35%)	13 (24.53%)	5 (27.78%)			
Swallow	6 (8.45%)	2 (3.77%)	4 (22.22%)			
Speech and Swallow	9 (12.68%)	7 (13.21%)	2 (11.11%)			
Without	38 (53.52%)	31 (58.49%)	7 (38.89%)			
**Education level**				Fisher test	0.9056	-
Primary School	4 (5.63%)	3 (5.66%)	1 (5.56%)			
Middle School	23 (32.39%)	15 (28.30%)	8 (44.44%)			
High School	15 (21.13%)	13 (24.53%)	2 (11.11%)			
**Modified rankin scale**				Fisher test	0.8286	-
1	1 (1.41%)	1 (1.89%)	0 (0.00%)			
2	7 (9.86%)	5 (9.43%)	2 (11.11%)			
3	18 (25.35%)	15 (28.30%)	3 (16.67%)			
4	40 (56.34%)	28 (52.83%)	12 (66.67%)			
5	5 (7.04%)	4 (7.55%)	1 (5.56%)			

Description of demographic data and characteristics of disease [Mean (SD) or count (percent)]. Gender differences were estimated by t-test for continuous data and χ^2^ test for categorical data.

There were no significant differences in education level (*p* = 0.9056), stroke types (*p* = 0.6515), involved side (*p* = 0.3757), age (*p* = 0.2978), the course of disease (*p* = 0.4693), concomitant disorder (*p* = 0.1007), and scores of MBI (*p* = 0.9818) across gender.

### Data processing

#### Data shaping

**Figure 2:**
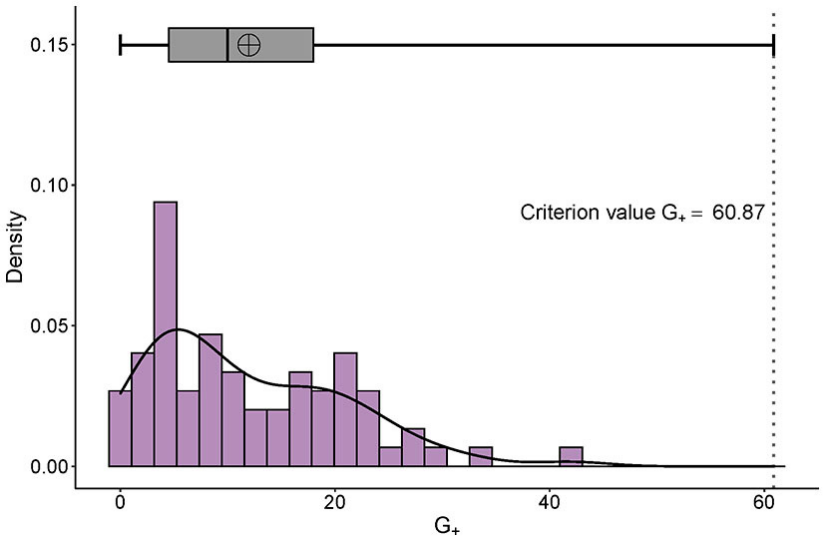
Distribution of G+ and its adjusted boxplot. The right-skewed distribution of personal G+ can be seen. The x-axis is the number of the G+ and the y-axis represents the probability density. The adjusted criterion value G+ is 60.87.

There was a significant but weak correlation between the number of Gutmann errors and the total score of the binarized M-SSNS data (*p* = 0.011, Spearman correlation coefficient = −0.30, CI 95% [−0.50, −0.06]) (Akoglu, 2018). All participants demonstrated G_+_ below the extreme boundary and qualified for further analysis (Figure 2, adjusted criterion value G_+_ = 60.87).

#### Scale formation

According to the scalability analysis, H = 0.2109, standard error = 0.0447. Since H < 0.3, 19 items were required for further screening (Sijtsma and van der Ark, 2017). By utilizing the AISP, the item distributions with different lower bounds of scalability were shown in Table 2. The recommended bound value of 0.42 detected five (26.32%) unscalable items. They were C4, R1, R2, WN2, and WN3. The remaining 14 items of M-SSNS were chosen for further IRT modeling. There were two models (dim1_3PL and dim2_3PL in Table 3) with good fitness (*p* < 0.05, RMSEA < 0.1, CFI and TLI > 0.9). The two models did not differ significantly (*p* = 0.0604 > 0.05, χ^2^ = 21.6895, df = 13). The AIC values of dim1_3PL and dim2_3PL were 979.90 and 984.21, respectively. The sample size adjusted AIC values of dim1_3PL and dim2_3PL were accordingly 1,108.90 and 1,394.87. Thus, the unidimensional 3PL model was selected based on the lower AIC. The three parameters were difficulty, discrimination, and pseudo-guessing.

**Table 2:** Item distributions with different lower bounds of scalability.

**Lower bound**	**Max scale**	**Scalable items (%)**	**Unscalable items (%)**
0.30	3	15 (78.95%)	4 (21.05%)
0.33	2	13 (68.42%)	6 (31.58%)
0.36	3	15 (78.95%)	4 (21.05%)
0.39	3	14 (73.68%)	5 (26.32%)
0.42	4	14 (73.68%)	5 (26.32%)
0.45	3	12 (63.16%)	7 (36.84%)
0.48	3	12 (63.16%)	7 (36.84%)
0.51	3	12 (63.16%)	7 (36.84%)
0.54	4	13 (68.42%)	6 (31.58%)

The number (%) of scalable items and unscalable items is listed according to the varied lower bounds and maximal scale.

**Table 3:** Goodness of fit test by using M2 for candidate models.

**Model**	**M2**	***df***	***p***	**RMSEA [95% CI]**	**TLI**	**CFI**
dim1_Rasch	213.4810	90	0.0000	0.1400 [0.1151, 0.1632]	0.5118	0.5172
dim1_2PL	122.3145	77	0.0008	0.0917 [0.0591, 0.1206]	0.7906	0.8228
dim1_3PL	77.9232	63	0.0976	0.0582 [0.0000, 0.0963]	0.9157	0.9417
dim1_4PL	76.4557	49	0.0073	0.0895 [0.0468, 0.1260]	0.8006	0.8926
dim2_2PL	90.4270	64	0.0165	0.0768 [0.0339, 0.1107]	0.8531	0.8967
dim2_3PL	62.8479	50	0.1049	0.0606 [0.0000, 0.1024]	0.9086	0.9498
dim2_4PL	61.4755	36	0.0051	0.1005 [0.0546, 0.1415]	0.7482	0.9004

The “dim” represents the dimension (1 or 2). The “PL” stands for the number of parameters, including difficulty, discrimination, pseudo-guessing parameter, and careless responding, respectively. RMSEA is the root-mean-square error analysis with a 95% confidence interval. CFI stands for the comparative fitting index. TLI represents the Tuck-Lewis index.

#### Local independence

**Figure 3:**
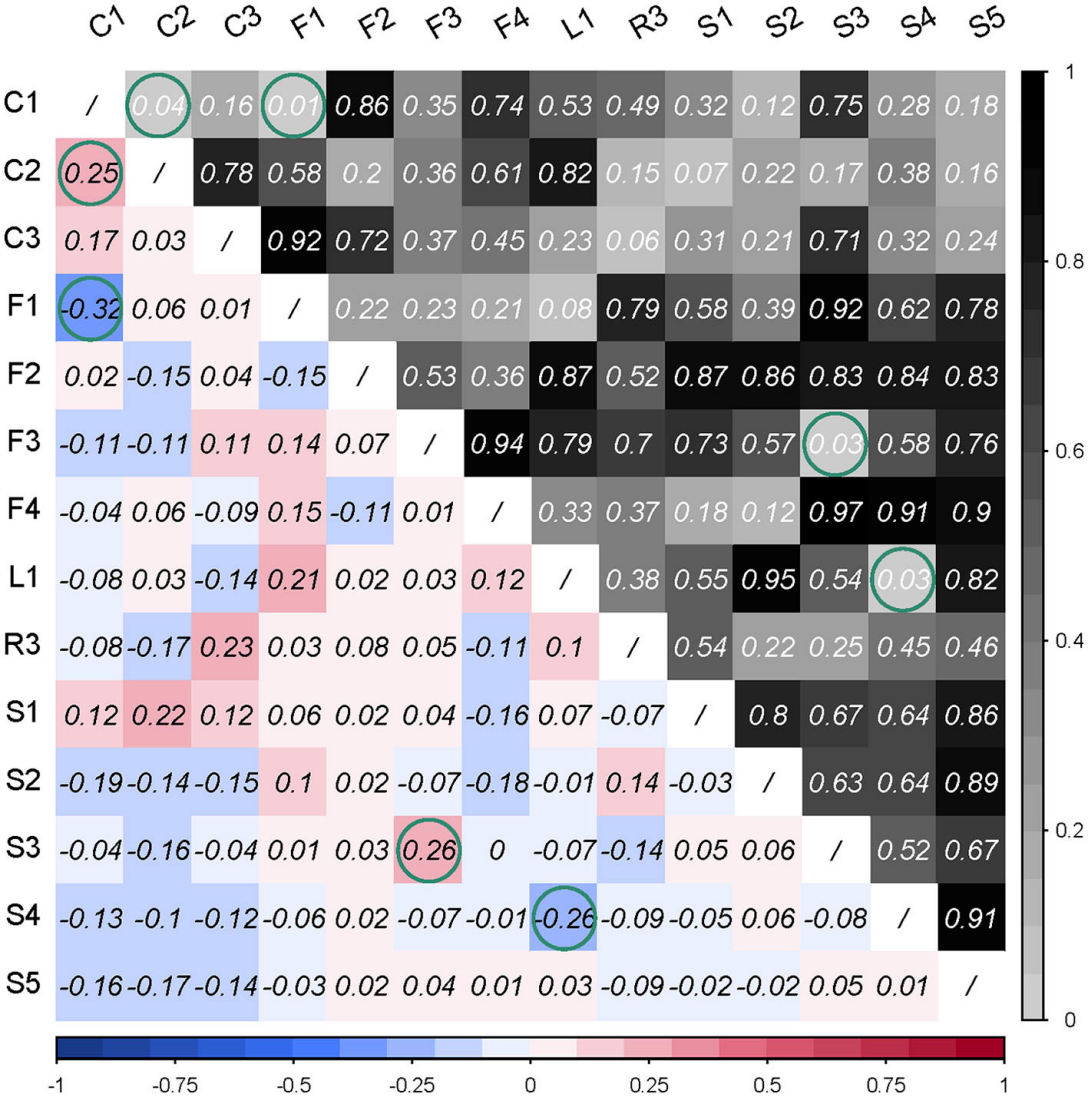
Pearson chi-square test of residuals was used for checking the local dependency. The triangle in the right upper corner reveals the *p*-value of the Pearson's chi-square test, and the triangle in the left lower corner is the Cramer's V value of correlation strength. Green circles are marked with significant correlation (*p* < 0.05) or strong correlation (Cramer's V > 0.15).

As can be reviewed from Figure 3, there were four item pairs with significantly (*p* < 0.05) strong correlations (absolute value of Cramer's V > 0.15). The positively correlated pairs were C1 and C2, and S3 and F3. The negatively correlated pairs were C1 and F1, and L1 and S4. Considering these pairs simply occupied little proportion of total pairs, they were kept in the scale.

#### Item fitness

Table 4 exhibited that C3 and S2 were poorly fitted at α = 0.05. The difficulty and discrimination values of C1 “*Do you have a son or daughter”* were highly similar to C3 “*In the past month, how often were you in contact with your children by telephone, WeChat (Wēixìn), Email or other ways?”* The discrimination of C1 vs. C3 was 59.4142 vs. 60.4686 and the difficulty was 0.6237 vs. 0.6334. However, pseudo-guessing of C3 (0.3139) was lower than C1 (0.8278). Since C1 also determined whether C3 applied, C3 was kept in the scale. The discrimination of S2 “*Are you satisfied with the frequency of contact with relatives?”* was 2.1670, the difficulty was −0.8520, and the guess degree was 0. In the data shaping process, item R1 “*How many close relatives do you have?”* has been removed. Given the relatives' relationship as an important interpersonal relationship especially for Chinese people, S2 was still considered to be retained in the final scale. Therefore, considering the cultural background and clinical application, C3 and S2 were employed at α = 0.01.

**Table 4:** Fitness and three parameters of each item.

**Item**	**Difficulty**	**Discrimination**	**Guessing**	**RMSEA**	***S*** **−*X*^2^**	***df***	***p***	***Adjusted p***
C1	0.6237	59.4142	0.8278	0.0000	4.4958	8	0.8099	0.9470
C2	1.9006	8.0072	0.4338	0.1033	13.9748	8	0.0824	0.3220
C3	0.6334	60.4686	0.3139	0.1615	22.6054	8	0.0039	0.0331
F1	0.6238	0.5297	0.0001	0.0000	5.2512	8	0.7304	0.9470
F2	3.2690	1.6753	0.0000	0.0000	4.0575	8	0.8519	0.9470
F3	1.1190	24.5189	0.1228	0.0000	2.4669	8	0.9633	0.9633
F4	1.7714	2.5044	0.0728	0.0000	3.7451	8	0.8794	0.9470
L1	0.1485	3.1670	0.7445	0.0207	8.2410	8	0.4103	0.7180
R3	0.6374	1.2356	0.0000	0.0874	12.2799	8	0.1391	0.3766
S1	0.2725	8.0239	0.7445	0.0000	6.2236	8	0.6222	0.9470
S2	−0.8520	2.1670	0.0000	0.1587	22.1059	8	0.0047	0.0331
S3	−0.3914	3.0233	0.0000	0.0371	8.7707	8	0.3620	0.7180
S4	−0.1682	50.2735	0.1431	0.1002	13.6273	8	0.0920	0.3220
S5	−0.2518	65.3930	0.0000	0.0821	11.7773	8	0.1614	0.3766

RMSEA represents the approximate root-mean-square error. The S-X2 is the IRT statistic derived from Pearson χ^2^ test, which is approximate to the χ^2^ distribution. The adjusted p-value of item fitness was calibrated by Benjamini-Hochberg multiple test. Item fitness was good if the adjusted p > 0.05.

### Reliability and validity of the SSNS

**Figure 4:**
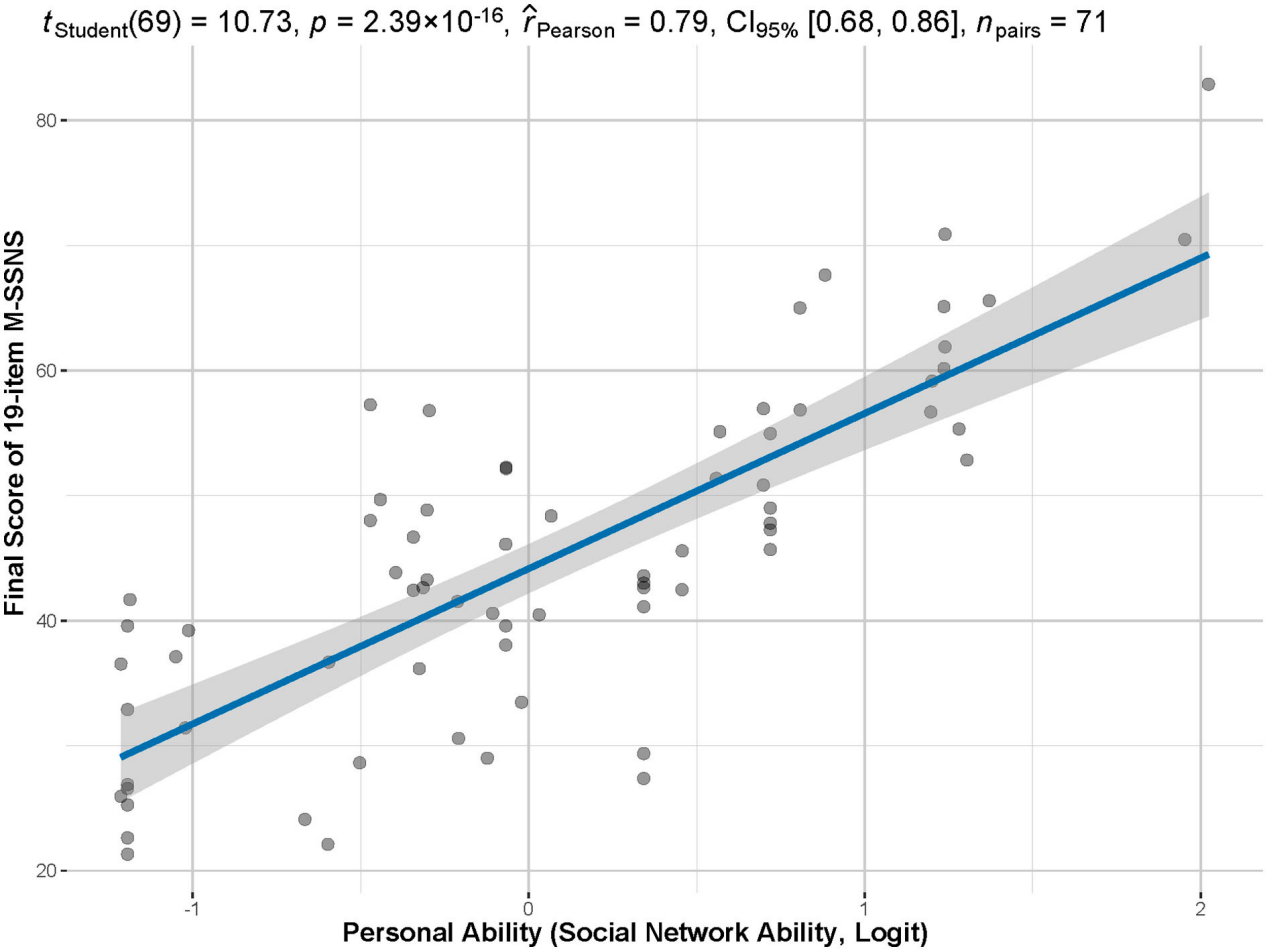
Correlation between total scores and personal abilities. The total score of the M-SSNS has a significant and strong correlation between the individual competency estimated by the model (in logit) and the actual total score of the scale (*p* < 0.001, *r* = 0.79).

The Cronbach's α of the 14-item scale was 0.7192 with the Guttman's λ_2_ = 0.7567, Molenaar Sijtsma ρ = 0.7491, and LCRC= 0.8657, indicating that the 14-item had great internal consistency. Figure 4 showed that the estimated individual social competence by the 14-item IRT model was highly related to the final score of the 19-item M-SSNS (*p* = 2.39 × 10^−16^ < 0.001, *r* = 0.79), indicating the individual social ability calculated by the model could reflect the score of M-SSNS.

**Figure 5:**
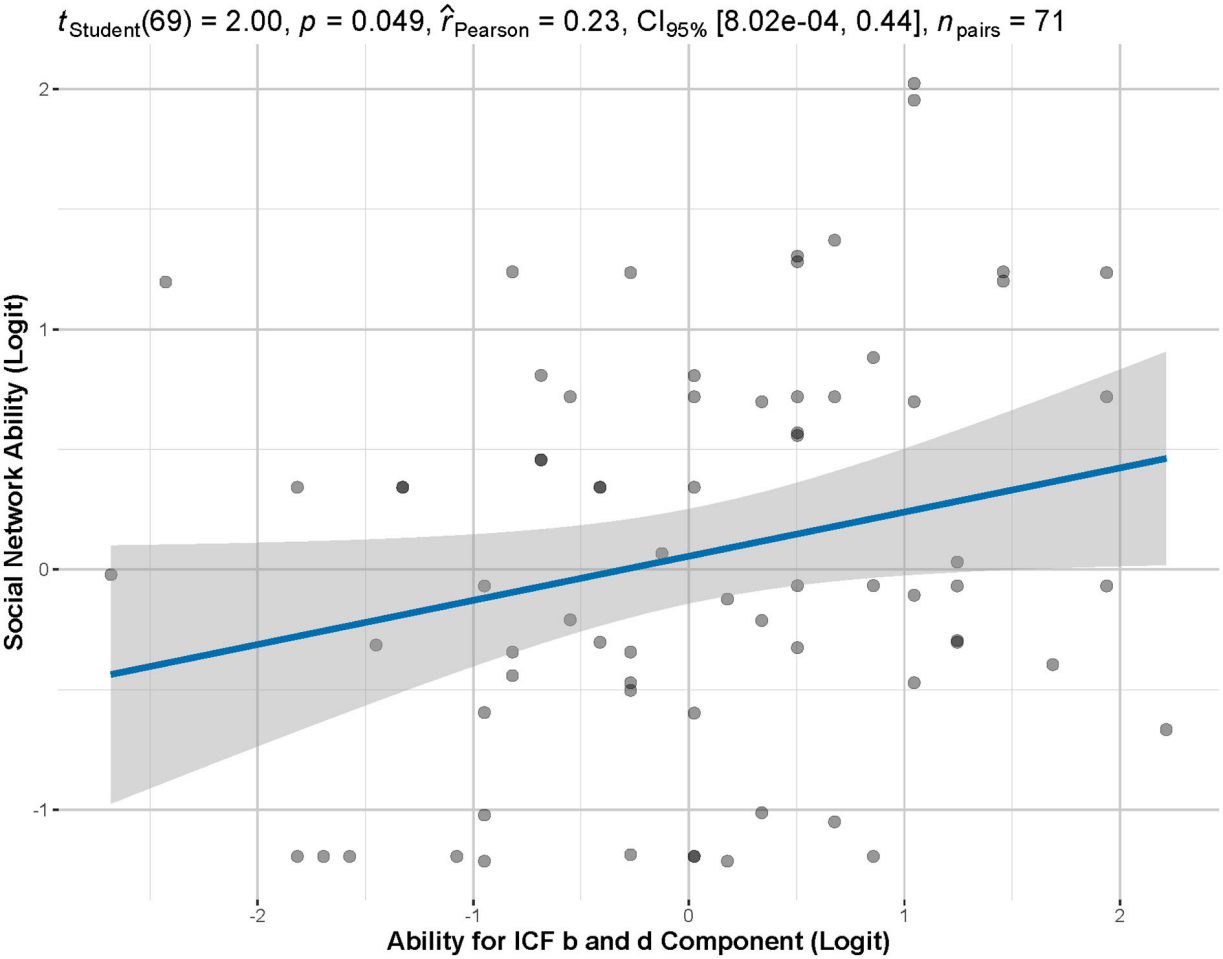
Correlation between M-SSNS-IRT-based personal ability and ICF-IRT-based functional ability. According to Pearson's correlation analysis, there is a significant but weak correlation between functional ability and individual social ability (*p* = 0.049, *r* = 0.23).

To get comprehensive results, the scale was derived from the two domains of body function, and activity and participation in the extended ICF Core Set for Stroke (Glässel et al., 2012). ICF-IRT-based personal functional status was weakly correlated with the social ability (*p* = 0.049, *r* = 0.23), as shown in Figure 5.

### Application of social competence evaluation in stroke individual

**Figure 6:**
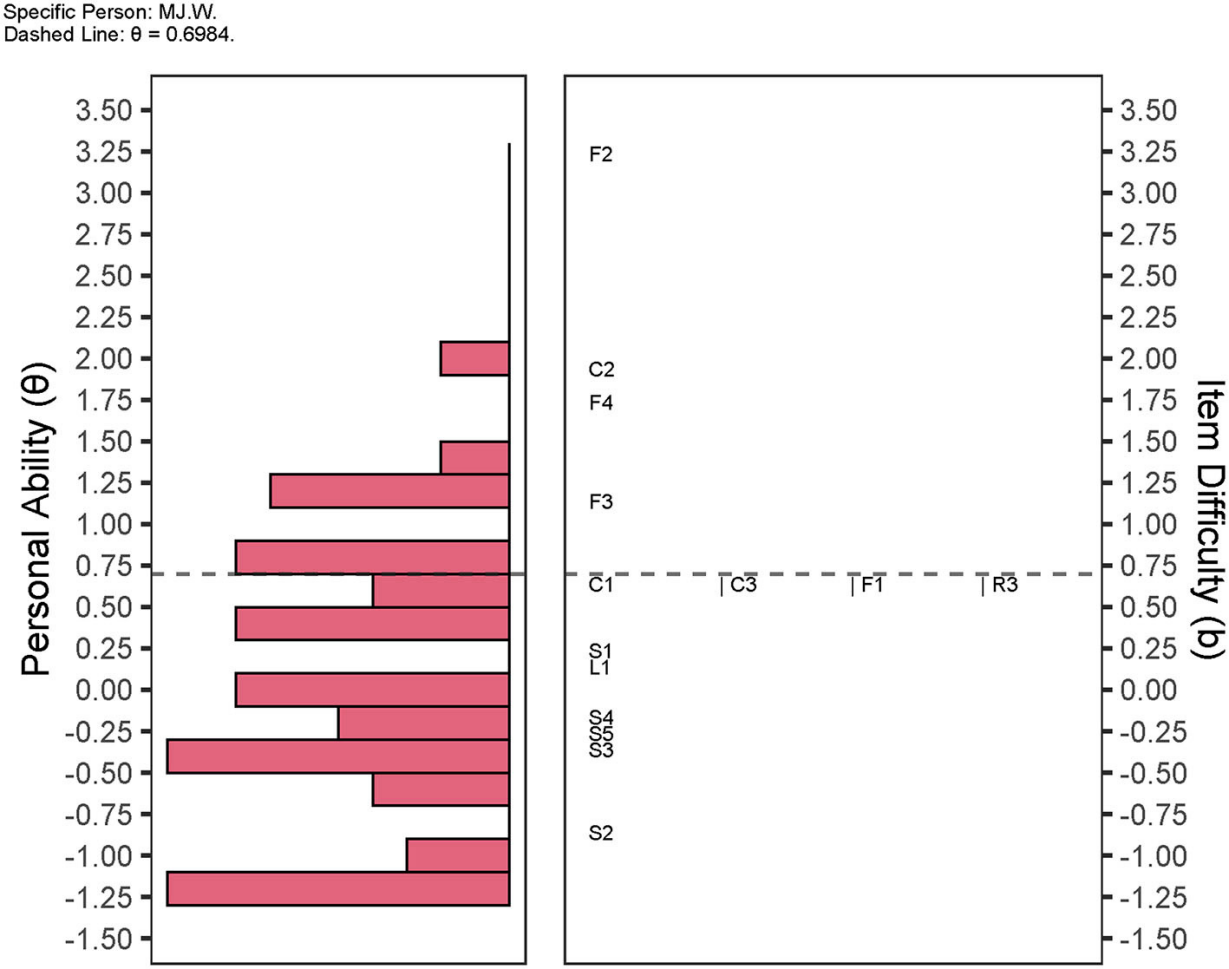
Wright map of personal social ability and item difficulty. The distribution of individual social competence is demonstrated on the left histogram. The right side manifests the distribution of item difficulty. The wright map units the personal ability and item difficulty with logit. As an example, the social competence for patient sh.sub.22 (θ = 0.6984) are plotted as horizontal dashed lines. Items F3, F4, C2, and F2 are above the dashed line. Items C1, C3, F1, and R3 are almost at the same level of difficulty level. S domains and L1 are below the dotted line.

Figure 6 demonstrated the personal ability in social involvement of patient 78 (MJ.W.). F2 “*How often did you get together with your friends in the past month”* was the most difficult item for MJ.W. to attain. F3, F4, and C2 were also difficult items.

## Discussion

This was the pilot study to investigate the application of the Mandarin version of SSNS and illustrate the IRT-based individual social competence among the stroke population in the Chinese mainland. The psychometric properties of the M-SSNS were also examined in this study based on the unidimensional 3PL IRT model (Table 4). The M-SSNS had excellent internal consistency and acceptable content validity. Furthermore, this scale could be used as a tool to assess the individual social ability of patients after stroke, calculated by the IRT (Figure 5).

We should emphasize the application of the IRT model of M-SSNS. Two critical values including personal ability and item difficulty can be achieved simultaneously in the IRT model. Personal ability implies the social competence or social ability among individuals with stroke in this study. Item difficulty indicates the scoring probability of an item among the individuals. Scores of single-item responses ≥50 were accordingly set as 1 (Satisfactory) and those with a score < 50 were considered as 0 (Dissatisfactory). The more difficult the item is, the more likely patients rated response options as “no children,” or “no child/friend/relatives' visits in person or *via* the telecommunication tools within 1 month,” which should be counted as 0 (Dissatisfactory). Given the individual social competence and challenging items (Figure 6), targeted interpersonal consulting, education, and advice could be applied in clinical practice.

### Correlation between ICF-based functional status and IRT-based social competence

Post-stroke physical limitations could restrain patients from an active social life. Anxiety after stroke also hampers functional recovery, diminishes the quality of life (Li et al., 2019), and can lead to discomfort in social interactions (Chun et al., 2018). Social barriers keep patients from social engagement rather than their individual impairments. This can include issues, such as inaccessible information and service which would engage them in inauthentic inclusion in organizational procedures and practices (Urimubenshi and Rhoda, 2011). Social ties can still drastically decrease, even when physical recovery is complete. Thus, patients after stroke are inevitably exposed to a high risk of participation limitations and difficulties in social reintegration (Kapoor et al., 2017). We compared individual functioning levels estimated by the IRT model of ICF b and d components with personal social competence measured by the IRT model of M-SSNS. Although we found that they are significantly related to each other, the correlation strength is weak (Figure 5). It may indicate functional assessments solely based on activity limitations and participation restrictions can capture, but may be unable to fully reflect, problems encountered by stroke survivors (Kapoor et al., 2017). The M-SSNS offers a complementary tool for ICF-based functional assessment.

### Perspectives between clinical practices and IRT-based social competence

The concept of social participation usually means engagement in activities with families, friends, peers, and the community. Participation restrictions involve inabilities to return to the previous life, as well as a lack of social interactions (Urimubenshi, 2015). Successful social participation likely has an array of meanings and affects different people differently. These differences could include culture, customs, and individuals' expectations. Following a stroke, patients may be living with some form of disability for the rest of their lives. Previous studies have further indicated that social support has a positive influence on chronic disease recovery. Foley et al. (2019) point out that higher perceived social support during post-stroke rehabilitation may enhance social participation. On the contrary, due to social isolation, physical health can also be affected and survivors are prone to develop other health conditions (Holt-Lunstad, 2018). How can we better prepare them to regain personal social connections and reconstruct their social network?

Here, we took patient 78 (MJ.W.) as an example (Figure 6). He is a retired teacher aged 70 years. The F2, F4, and F3 related to the friend domain were considered the three difficult items for MJ.W. The C2 “*In the past month, how often did you see your children?”* was also a difficult item for this patient to achieve. As a family-oriented country, family members are extremely cohesive in China (Epstein et al., 2012). Chinese people are more likely to seek emotional and financial support from families rather than friends (Chan et al., 2012).

When we consider this phenomenon, we cannot avoid mentioning the age of the subjects in our study (62.96 ± 12.62). These older generations usually have great connections with their siblings, cousins, and other family members, and maintain great relationships with their neighbors. Since 1970, people started moving from small Chinese single-story homes to apartments with rapid population growth and social and economic developments. The relationship between neighborhood and community also became gradually distant. People were not allowed to have more than one baby following the national policy of one child in 1980 (Feng et al., 2016). Although adult children are also an important resource to provide care for parents, it has been difficult for them to keep a full-time job while taking care of their sick parents who are hospitalized. The middle generation also needs to juggle time to look after their offspring who may be still in primary school. Patients' spouse or siblings who already retired from work have more time to visit patients. As a result, seeing your own child became a very difficult item for this patient.

Nevertheless, social connection and structure dramatically changed during the COVID-19 pandemic (Podury et al., 2021). People are experiencing burnout and deprivation of social life during frequent lockdown and work at home. The 48 or 72-h negative COVID-19 testing result is required before visiting someone in the hospital. Thus, access to inpatient visits has become significantly limited.

Moreover, Figure 6 may provide insights into clinical practices. Although the MJ.W. had difficulty seeing children and losing connection with friends (see the F2, F3, F4, and C2 in Figure 6), it was easy for this patient to be satisfied with personal social relationships (see the L and S-items in Figure 6), and the item with relatively little difficulty was the F3 “*In the past month, how often were you in contact with your close friends by telephone, WeChat (Wēixìn), Email or other ways?*” Therefore, if we want to improve individual social competence, the F3-oriented interventions should be applied, such as helping close friends contact the patient *via* telecommunication tools.

### Study limitations

This study has several limitations. First, the subjects were recruited from an inpatient rehabilitation hospital. Hence, the representativeness of the sample population may not be sufficient. The outpatient population will also be included to achieve a better generalization of the study results in the future. Second, the sample size should be expanded to further validate the result presented in this study. Third, further research is needed to advance our understanding of environmental factors after we expand our subject numbers and the heterogeneity of these patients after stroke. Although the effect of COVID-19 is not under human control, the study result should be reconfirmed after the pandemic.

## Conclusion

A 14-item dichotomous IRT model with confident reliability and validity can be constructed from a 19-item M-SSNS. This model offers estimation for personal social competence and item difficulties and provides new insight for conducting interpersonal relationship consulting, education, and advice. Further study is still required to consider the environmental influence on patients' willingness to engage in their social surroundings.

## Data availability statement

The datasets presented in this article are readily available. Requests to access the datasets should be directed to the corresponding author.

## Ethics statement

The studies involving human participants were reviewed and approved by the Ethics Committee of The First Rehabilitation Hospital of Shanghai (IRB# YK-2021-02-011). The patients/participants provided their written informed consent to participate in this study.

## Author contributions

FL contributed to the research concept, supervised the entire study, performed the analysis, and generated the images. CF, Q-LL, and FL designed the protocol, recruited subjects, and wrote the manuscript with AF. Q-LL, FL, CF, and AF contributed to translating and adapting the original Stroke Social Network Scale to the Mandarin version. All authors contributed to the article and approved the submitted version.

## Funding

This study was funded by the National Nature Science Foundation of China (No. 81672255).

## Conflict of interest

The authors declare that the research was conducted in the absence of any commercial or financial relationships that could be construed as a potential conflict of interest.

## Publisher's note

All claims expressed in this article are solely those of the authors and do not necessarily represent those of their affiliated organizations, or those of the publisher, the editors and the reviewers. Any product that may be evaluated in this article, or claim that may be made by its manufacturer, is not guaranteed or endorsed by the publisher.
