# Supplementary material for: Mandarin Stroke Social Network Scale and Item Response Theory
Source: Front Stroke. 2022 Aug 11;1:903289. doi: 10.3389/fstro.2022.903289 (PMC12802792; doi:10.3389/fstro.2022.903289)
Supplement: Supplementary file 1 [file Table_1.docx]

|  | **Item** | **Response options** | **项目** | **回答选项** |
| --- | --- | --- | --- | --- |
| **C1** | Do you have any sons or daughters? | 0 = no children; 50 = sons; 100 = sons and/or daughters | 您有儿子或女儿吗？ | 0 = 没有；50 = 有儿子；100 =有儿子和/或女儿 |
| **C2** | In the past month, how often did you see your children? | 0 = not at all; 20 = about once a month; 40 = 2 or 3 times a month; 60 = at least once a week; 80 = 2 or 3 times a week; 100 = every day | 在过去的一个月，您经常见到您的子女吗？ | 0 = 没有；20 = 约一个月1次；40 = 一个月2-3次；60 = 至少一周1次；80 = 一周2-3次；100 = 每天 |
| **C3** | In the past month, how often were you in contact with your children by telephone, WeChat (*Wēixìn*), Email or other ways? | 0 = not at all; 20 = about once a month; 40 = 2 or 3 times a month; 60 = at least once a week; 80 = 2 or 3 times a week; 100 = every day | 在过去的一个月，您经常打电话、发微信，电子邮件或通过其他方式和子女联系吗？ | 0 = 没有；20 = 约一个月1次；40 = 一个月2-3次；60 = 至少一周1次；80 = 一周2-3次；100 = 每天 |
| **C4** | How far away does your nearest child or close relative live? | 0 = 50+ miles (or no child/relative); 25 = 16-50 miles; 50 = 6-15 miles; 75 = 1-5 miles; 100 = same house | 住所距离您最近的孩子或亲戚有多远？ | 0 = 大于100公里 (或没有子女/亲戚)；25 = 30-50公里；50 = 10-30 公里；75 = 2-9 公里；100 = 同一栋房子 |
| **F1** | How many close friends do you have? * | 0 = no friends; 14 = 1 friend; 36 = 2 friends; 43 = 3 friends; 57 = 4 friends; 71 = 5 friends; 86 = 6 friends; 100 = 7 or more friends | 您有多少亲近的朋友？ | 0 =没有；14 = 1个；36 = 2个；43 = 3个；57 =4个；71 = 5个；86 = 6个；100 ≥ 7个 |
| **F2** | In the past month, how often did you see your close friends? | 0 = not at all; 20 = about once a month; 40 = 2 or 3 times a month; 60 = at least once a week; 80 = 2 or 3 times a week; 100 = every day | 在过去的一个月里，您经常和朋友相聚吗？ | 0 = 没有；20 = 约一个月1次；40 = 一个月2-3次；60 = 至少一周1次；80 = 一周2-3次；100 = 每天 |
| **F3** | In the past month, how often were you in contact with your close friends by telephone, WeChat (*Wēixìn*), Email or other ways? | 0 = not at all; 20 = about once a month; 40 = 2 or 3 times a month; 60 = at least once a week; 80 = 2 or 3 times a week; 100 = every day | 在过去的一个月里，您经常打电话、发微信，电子邮件或通过其他方式和和朋友联系吗？ | 0 = 没有；20 = 约一个月1次； 40 = 一个月2-3次；60 = 至少一周1次； 80 = 一周2-3次；100 = 每天 |
| **F4** | How many of your close friends live nearby? (within 5 miles) | 0 = none of them/ no friends; 33 = some of them; 67 = most of them; 100 = all of them | 您亲密的朋友中有多少人住在附近？（10公里之内） | 0 = 没有/没有朋友；33 = 一些；67 =大部分；100 = 全部 |
| **L1** | How often do you feel lonely? | 0 = lonely all the time; 25 = lonely most of the time; 50 = lonely some of the time; 75 = lonely a little of the time;100 = never lonely | 您经常感到孤独吗？ | 0 = 总是感到孤独； 25 = 经常感到孤独；50 = 有时感到孤独； 75 = 很少感到孤独；100 = 从不觉得孤独 |
| **R1** | How many close relatives do you have?* | 0 = no relatives; 17 = 1 relative; 33 = 2 relatives; 50 = 3 relatives; 67 = 4 relatives; 83 = 5 relatives; 100 = 6 or more relatives | 你有多少亲近的亲戚？ | 0 = 没有； 17 =1个； 33 =2个；50 = 3个； 67 = 4个；83 = 5 个；100 ≥6个 |
| **R2** | In the past month, how often did you see your relatives? | 0 = not at all; 20 = about once a month; 40 = 2 or 3 times a month; 60 = at least once a week; 80 = 2 or 3 times a week; 100 = every day | 在过去的一个月里，您经常和亲戚相聚吗？ | 0 = 没有； 20 = 约一个月1次；40 = 一个月2-3次； 60 = 至少一周1次； 80 = 一周2-3次；100 =每天 |
| **R3** | In the past month, how often were you in contact with your relatives by telephone, WeChat (*Wēixìn*), Email or other ways? | 0 = not at all; 20 = about once a month; 40 = 2 or 3 times a month; 60 = at least once a week; 80 = 2 or 3 times a week; 100 = every day | 在过去的一个月里，您经打电话、发微信、电子邮件或通过其他方式和亲戚联系吗？ | 0 = 没有； 20 = 约一个月1次；40 = 一个月2-3次； 60 = 至少一周1次； 80 = 一周2-3次；100 =每天 |
| **S1** | How satisfied were you with the frequency of contact with your children? | 0 = very dissatisfied; 20 = fairly dissatisfied; 40 = a little dissatisfied; 60 = a little satisfied; 80 = fairly satisfied; 100 = very satisfied | 您对于与子女的联系频率满意吗？ | 0 = 非常不满意； 20 = 中度不满意； 40 = 一点点不满意；60 = 一点点满意；80 = 中度满意；100 = 非常满意 |
| **S2** | How satisfied were you with the frequency of contact with yourrelatives? | 0 = very dissatisfied; 20 = fairly dissatisfied; 40 = a little dissatisfied; 60 = a little satisfied; 80 = fairly satisfied; 100 = very satisfied | 您对于与亲戚的联系频率满意吗？ | 0 = 非常不满意； 20 = 中度不满意； 40 = 一点点不满意；60 = 一点点满意；80 = 中度满意；100 = 非常满意 |
| **S3** | How satisfied were you with the frequency of contact with your close friends? | 0 = very dissatisfied; 20 = fairly dissatisfied; 40 = a little dissatisfied; 60 = a little satisfied; 80 = fairly satisfied; 100 = very satisfied | 您对于与朋友的联系频率满意吗？ | 0 = 非常不满意； 20 = 中度不满意； 40 = 一点点不满意；60 = 一点点满意；80 = 中度满意；100 = 非常满意 |
| **S4** | How satisfied were you with the frequency of contact with neighbours? | 0 = very dissatisfied; 20 = fairly dissatisfied; 40 = a little dissatisfied; 60 = a little satisfied; 80 = fairly satisfied; 100 = very satisfied | 您对于与邻居的联系频率满意吗？ | 0 = 非常不满意； 20 = 中度不满意； 40 = 一点点不满意；60 = 一点点满意；80 = 中度满意；100 = 非常满意 |
| **S5** | How satisfied are you overall with your social network? | 0 = very dissatisfied; 20 = fairly dissatisfied; 40 = a little dissatisfied; 60 = a little satisfied; 80 = fairly satisfied; 100 = very satisfied | 您如何评价您的社交网络的整体满意度？ | 0 = 非常不满意； 20 = 中度不满意； 40 = 一点点不满意；60 = 一点点满意；80 = 中度满意；100 = 非常满意 |
| **WN2** | How many groups do you belong to? | 0 = no groups; 33 = 1 group; 67 = 2 groups; 100 = 3 or more groups | 您参加多少个团体组织？ | 0 = 没有； 33 = 1个； 67 = 2个；100 ≥3个 |
| **WN3** | How active were you in these groups? | 0 = don’t belong to any groups; 33 = belong but not active; 67 = fairly active; 100 = very active | 您积极参加这些团体/俱乐部活动吗？ | 0 = 不属于任何团体；33 = 是成员但不积极；67 = 中度积极； 100 = 非常积极 |

* close friends/relatives (not including children or spouse) are people you feel at ease with and can talk about what is on your mind?

* 关于亲密的朋友/亲戚的定义：感觉与之相处融洽轻松并能倾诉衷肠的人。
